# Pre-Impact Fall Detection with CNN-Based Class Activation Mapping Method

**DOI:** 10.3390/s20174750

**Published:** 2020-08-22

**Authors:** Jingyi Shi, Diansheng Chen, Min Wang

**Affiliations:** 1 Institute of Robotics, School of Mechanical Engineering and Automation, Beihang University, Beijing 100191, China; sannystone@buaa.edu.cn; 2 Beijing Advanced Innovation Center for Biomedical Engineering, Beihang University, Beijing 100191, China; 3 School of General Engineering, Beihang University, Beijing 100191, China; wmin@buaa.edu.cn

**Keywords:** fall detection, pre-impact, neural network, threshold-based, IMU

## Abstract

In this paper, we report our improvement on the prediction accuracy of pre-impact fall detection by applying a learning-based method on the real-time data from an IMU (inertial measurement unit)-sensor mounted on the waist, making it possible to achieve a high accuracy on a wearable device with the extracted features. Using the fixed threshold method is difficult for achieving satisfactory detection accuracy, due to various characteristics and behaviors in the movement of different individuals. In contrast, one could realize high-accuracy detection with machine learning-based methods, but it is difficult to apply them in the wearable devices due to the high hardware requirement. Our method merges the two methods above. We build a convolutional neural network (CNN) with a class activation mapping (CAM) method, which could highlight the class-specific region in the data and obtain a hot map of the fall data. After training on the MobiAct dataset, the model could achieve high-accuracy detection (95.55%) and obtain the region with high contributions to the classification. Then, we manually extract effective features and characteristics of this region and form our special threshold method, achieving pre-impact fall detection in real-world data. Consequently, our method achieves accuracy of 95.33% and a detection time of within 400 ms.

## 1. Introduction

Falls are a major threat to people’s health, since they are the cause of disability and injury-related deaths, especially for the elderly. The injuries caused by falls are serious, including fractures, joint dislocations, and cognitive decline, which pose high costs to both family and public health care. In order to avoid these bad consequences, pre-impact fall detection can significantly reduce complications by providing other auxiliary protection for fall victims, based on the early detection of falls, before hitting the ground. Thus, the pre-impact fall detection needs to leave relatively long lead-time for activating the protection.

Pre-impact fall detection is the process in which the tendency of a fall is about to happen, usually within a time frame of 200–400 ms [1], the time from a person’s imbalance to impact. Accelerometer-based fall detection systems are widely used in all fall stages, including pre-impact, impact, and post-impact [2]. However, it is difficult to distinguish human activities by only using a single acceleration sensor, because different human activities may generate similar acceleration data [3]. Therefore, some studies used a gyroscope along with an accelerometer integrated as a portable inertial sensor, whose data contains posture information, to detect a fall before impact [4]. In general, these pre-impact fall detection systems either use a threshold-based [5] or a machine learning-based method [6].

The threshold-based method checks if the value of handcrafted feature exceeds a pre-defined threshold at a certain frame, which is the simplest and lowest computational algorithm in fall detection [7]. Therefore, it can be widely used in wearable devices, due to the limited storage space and poor computation capacity of embedded systems. The handcrafted features and threshold value are manually determined based on the data collected from multiple imitative fall experiments, which requires researchers to have sufficient knowledge of human activity characteristics. However, because of the interclass variability, as well as interclass similarity among activities of daily living (ADLs) and falls, it is difficult to define the threshold value [8]. Furthermore, since the threshold depends on the experimental data, when the differences between real-world and experimental conditions are large, the detection accuracy reduces, while the false alarm rate increases. In our study, we exploit the class activation map (CAM) method to explore the data principle of fall detection, and perform threshold detection on the obtained feature regions. Our approach circumvents the difficulties mentioned above.

The machine learning-based method utilizes a pre-trained classifier to realize the automatic classification without manually setting the threshold value, so that the detection accuracy increases. Some machine learning-based methods still rely on the handcrafted features extracted from a sliding window on the data sequences as the input to the classifier, such as support vector machine (SVM) [9], decision tree, K-nearest neighbor (KNN), etc., which are collectively referred to as shallow learning. The features are mainly obtained by statistical analysis of the original data sequences in the time or frequency domain. One possible problem existing in shallow learning-based methods is that if the extracted features are not suitable enough, it will lead to the loss of important information laid in the original data, which will affect the final detection accuracy of the algorithm. Moreover, though more features sometimes help classify the falls and non-falls more accurately, the value calculation of massive features will be time-consuming, taking up too much running time of the algorithm.

To extract the effective features for identifying falls, some studies adopt deep learning-based methods, such as convolutional neural networks (CNNs) [10] and recurrent neural networks (RNNs), where a deep architecture with multiple layers is proposed for automating feature learning from the raw time series data. The algorithm first uses the sliding window to split the sensor data, and then the raw data can be directly used as the classification model input after a simple preprocessing. The convolutional network finds suitable data features through iterative learning of the training dataset, and finally outputs its classification (falls or non-falls) to achieve end-to-end processing. Since a CNN is good at discovering intricate structures in high-dimensional data, the deep learning-based method achieves high fall detection accuracy. However, the application of deep learning in fall detection based on wearable sensors has not been actually used, due to limited computational capacity and energy consumption of wearable devices. In our research, the CAM method [11] provides a way to visualize the class-specific region for the corresponding class, and to understand how the convolutional networks work for the setting of classification. Consequently, we can explore the data principle of fall detection. By combining threshold detection on the obtained feature region, the method can be applied to wearable devices with comparable accuracy.

**Figure 1:**
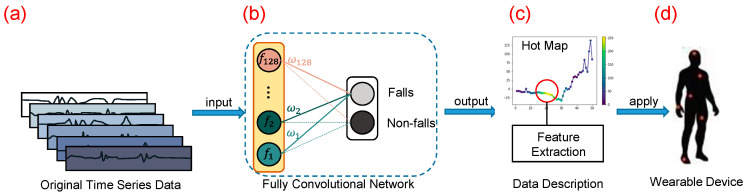
An overview of our proposed method. (**a**) The input of the convolutional neural network (CNN) is the original time series data. (**b**) We use CNN to train a high-accuracy pre-impact fall detection model. (**c**) Based on the class activation mapping (CAM) method, we obtain the hot map of the original time series, which highlights the contributing region. By analysis on this region in detail, we manually extract effective features and characteristics. (**d**) Combining threshold detection on the obtained feature region, the method can be applied to wearable devices with comparable accuracy.

To overcome the aforementioned challenges on feature extraction, threshold defining, and algorithm complexity, we proposed a pre-impact fall detection approach with a CNN-based class activation mapping method using wearable sensors, shown in Figure 1. This approach consists of:

- The use of a CNN to train a high accuracy pre-impact fall detection model, and its extension, the CAM, to highlight the class-specific region in the data. The contributing region is the basis of feature extraction.
- A manual statistics and description for data characteristics in the region to extract effective features. Based on the setting of classification working on a CNN, the features in the contributing region are more discriminative and suitable for distinguishing pre-impact falls from other human activities.

In summary, our contributions are three-fold:

1. A new pre-impact fall detection approach is proposed, combining the advantages of convolutional network-based and threshold-based methods, to achieve a high fall detection accuracy within a short time (400 ms) after fall start, and reduce the computational cost to run on the wearable device.
2. A feature extraction strategy is proposed to help researchers find the features in time series data, lessening the labor on manually handcrafted features.
3. Experimental verification on both public dataset and self-collected data.

## 2. Methods

**Figure 2:**
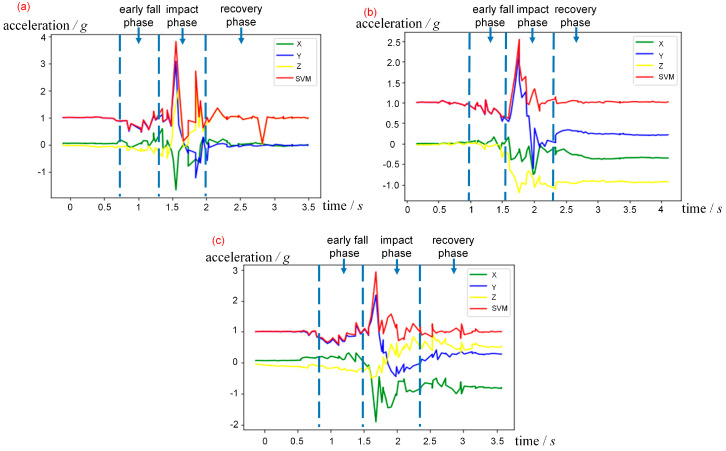
The acceleration data of the complete falling action. The x, y, z, and SVM labels represent x-axis, y-axis, z-axis, and sum vector magnitude of acceleration, respectively. (**a**) A fall backward sample. (**b**) A fall forward sample. (**c**) A fall laterally sample.

The inertial sensor data of three complete falling actions is shown in Figure 2. Fall action can be divided into three phases: early fall phase, impact phase, and recovery phase. It can be seen from the figures that the most obvious difference between falling and other daily actions is the maximum acceleration during the impact phase when the human body hits the ground. In order to achieve pre-impact fall detection, we want to extract the characteristics during the early fall phase. Since the ground support force is less than gravity when the human feet gradually leave the ground, the acceleration of the human body is less than gravitational acceleration, showing a slight weightlessness, while the characteristic of slight weightlessness can also be found in some daily actions, such as walking, running, and jumping. Therefore, we propose a network to validate that the fall data only containing the early fall phase is sufficient to distinguish it from other daily actions.

Although the network can successfully implement early detection, the learning-based method is hard to apply in wearable devices, due to its high computational cost. We hope to understand how the network works on detection, and what features the network has extracted. Additionally, the network not only detects pre-impact falls, but also highlights class-specific regions in the data. The backbone of the network is a one-dimensional CNN combined with the CAM method. We train our network on the specific dataset and obtain the early fall detection result. After that, we analyze the highlighting region of hot maps and find that the characteristics of the fall data during early the fall phase can effectively pre-detect a fall before impact.

### 2.1. Network Architecture

**Figure 3:**
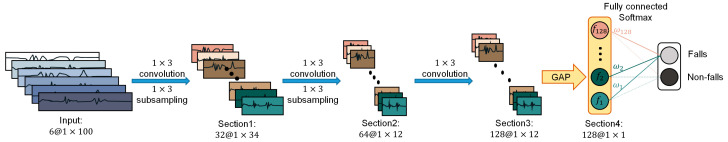
CNN network architecture: The network consists of three 1D convolutional layers with 32 output channels, 64 channels, and 128 channels, respectively, one global average pooling layer, one linear fully-connected layer, and one softmax layer. The input data dimension is (6, 100), and the output is the classification result of falls or non-falls.

We propose a CNN-based architecture, shown in Figure 3, which consists of three 1D convolutional layers with 32 output channels, 64 channels, and 128 channels, respectively, one global average pooling layer, one linear fully-connected layer, and one softmax layer. The convolutional layers are with (1, 3) filters and ReLu activation functions, which focus on the discriminative features for classification. The first two convolutional layers are followed by max pooling layers, while the last ones are not, in order to keep the data length for class activation mapping. The global average pooling (GAP) layer outputs the average of each channel at the third convolutional layer, and reduces the dimension of feature map from (1, 12) to (1, 1), which is followed by one single linear fully-connected. Finally, we use cross-entropy loss to judge classification quality, which guides training.

To detect a person’s pre-impact fall, we construct an input tensor of six channels, which consist of tri-axial acceleration and tri-axial angular velocity. Raw data are time series data acquired from an inertial sensor worn on the human body and recorded as nx6-dimension tensor. Since data come from multimodal sensors (accelerometer and gyroscope) which need to be fused, we use early fusion [12], where the filter’s first dimension of the first convolutional layer is equal to the number of input channels. After the first convolution, all channels are fused into one dimension.

### 2.2. Training

We demonstrated our network on MobiAct [13], which consists of falls and several ADL data, acquired by inertial sensors worn on the waist with a sampling rate of 200 Hz. Here, we used data of falls and frequent ADLs, including standing, walking, jumping, and jogging. Since the network requires input data with the dimension of (6, 100), we intercepted the corresponding region from each sample and formed our training dataset. In order to detect a pre-impact fall, instead of using complete fall data to train our fall detection model, we used short snippets of falls around the fall-start frame, and short snippets of ADLs as training samples. One great advantage of MobiAct is that the sensor data series is labeled at each time frame. We intercepted the original data in a time window of 200 length (the data in one second) after each activity-start frame and then subsampling to 100 to meet the input dimension.

We implemented our model in PyTorch. For all trainings, the model was trained with the Adam optimizer with a 0.0001 learning rate, a 2.3:1 training set (1047 samples) to test set (449 samples) ratio, and a 4 batch size. The training runs on a desktop with a 1060Ti card. There were 219 fall samples and 230 ADL samples in the test dataset. Our model correctly identified 207 (94.52%) fall samples and 222 (96.52%) ADL samples, achieving accuracy of 95.55%.

### 2.3. Class Activation Mapping Method

Many previous studies based on artificial intelligence (AI) algorithms mostly focused on the fall detection results, but lacked a description of the characteristics of the fall data. This means that fall detection is not fundamentally different from classification of other time series data. Due to that, we wanted to investigate the contribution of different segments of fall inertial data to the detection results, and we incorporated the CAM method. The CAM method was successfully used to highlight the class-specific region, which the network learned from the data, to sort different classes [14]. Namely, it could show the region that contributes to classification results. After the convolutional network was well-trained, we could visualize the class-specific region detected by convolutional networks to understand how the CNN works on classification, and what features the CNN had extracted. Through weighting the sum of the last convolutional layer, we can obtain a hot map of the contribution of the original data to the classification result.

**Figure 4:**
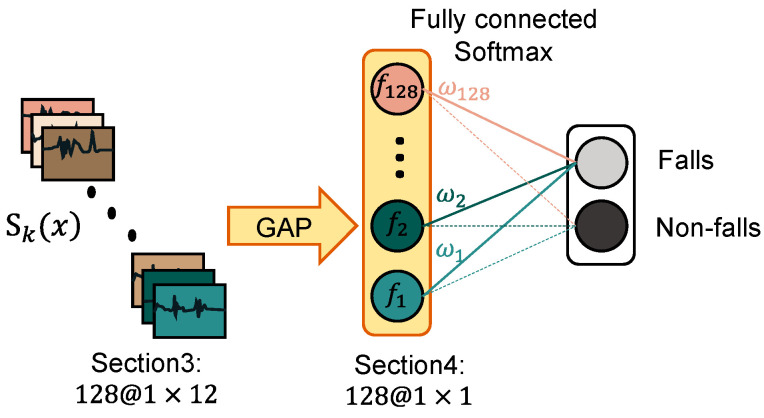
The network architecture that can apply the CAM method. The key layer is the global average pooling (GAP) layer, which establishes the mapping relationship between feature maps and classes. $S_{k}\left(x\right)$ represents the output sequence of the last convolutional layer on the channel k. $f_{k}$ is the output of channel k after the GAP.$\;\omega_{k}^{c}$ represents the weight of the features of each channel, k, to different class, c.

We obtained a discrete set of fall inertia data to describe the fall. However, we were not sure what interval data can best predict the occurrence of a fall. In this work, we exploited the CAM to explore this problem. The network architecture that can apply the CAM method is shown in Figure 4. For the input inertial sensor time series, let $S_{k}\left(x\right)$ represent the output sequence of the last convolutional layer on the channel k, and x represent the temporal location on the sequence. The output of channel k in the global average pooling layer can be expressed as $f_{k}=\sum_{x}S_{k}\left(x\right)$. $\omega_{k}^{c}$ represents the weight of the features of each channel k to different class c, then input of the final softmax function can be defined as $g_{c}$.

(1) $$\begin{array}{cc} g_{c} & =\underset{k}{\sum }\omega_{k}^{c}\underset{x}{\sum }S_{k}\left(x\right)\\ & =\underset{k}{\sum }\underset{x}{\sum }\omega_{k}^{c}S_{k}\left(x\right) \end{array}$$

Therefore, a class activation mapping from sequence to each class c can be established, defined as $M_{c}$.

(2) $$M_{c}=\underset{k}{\sum }\omega_{k}^{c}S_{k}\left(x\right)$$

$M_{c}\left(x\right)$ directly indicates how important the location x in the time series is for the sequence to be classified as c.

For visualization, the importance of the data contributing to fall detection was normalized and mapped to the range of [0,255]. In this way, the importance from low to high can be expressed as a color bar from purple to yellow. The length of the time series after three-layer convolution and pooling is generally inconsistent with the length of the original data input. To visualize the areas in the original data that contribute to the fall detection, the class activation map was upsampled, and the color was superimposed on the original data.

### 2.4. Pre-Impact Fall Characteristics Analysis

**Figure 5:**
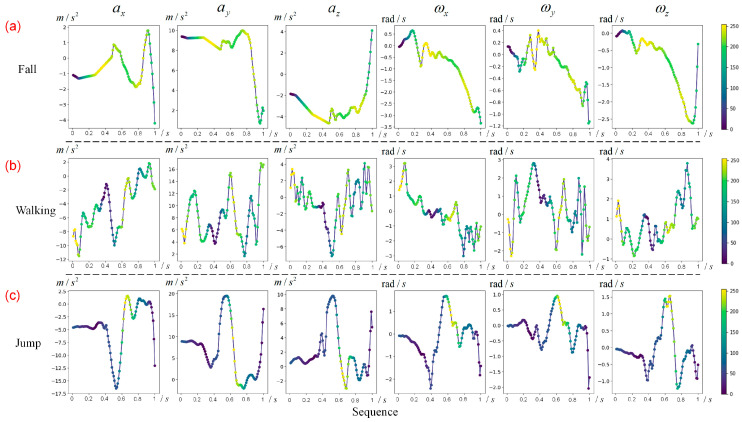
The hot map of four samples of the MobiAct dataset. The color bar indicates the importance of regions in the sequence for classification. $a_{x}$, $a_{y}$, and $a_{z}$ represent x-axis, y-axis, and z-axis of acceleration, respectively. $\omega_{x}$, $\omega_{y}$, and $\omega_{z}$ represent x-axis, y-axis, and z-axis of angular velocity, respectively. (**a**) One fall sample. (**b**) One walking sample. (**c**) One jump sample.

We obtained the hot map of each sample using the CAM method, as shown in Figure 5. Figure 5a shows the hot map of one fall sample, Figure 5b shows the hot map of one walking sample, and Figure 5c shows the hot map of one jump sample. In detail, the highlighting region (in yellow) in Figure 5a means that the most important feature of a fall is included here. The y-axis is the direction of gravitational acceleration. While falling, the sum vector magnitude (SVM) of tri-axial acceleration shows a decline. Finally, we come to the following three principles:

- The SVM at the beginning of fall action is slightly less than one gravitational acceleration. The slight decrease in SVM is an important feature of a fall, and this timestamp is defined as the start frame of a fall.
- The slight weightlessness over a period, shown as a gradual decline in SVM, can provide contributing information for the model to identify the fall, though the decline sometimes shows a fluctuation, as shown in Figure 5a. This period is defined as the early fall phase.
- The angular velocity remains relatively low during the early fall phase.

We test these principles in real-world experiments in the next section. Figure 5b,c demonstrate the important regions of walking and jumping, respectively. Intuitively, the contributing regions show huge differences from the ones of falls. Since they are not the focus of this study, we will not explore their characteristics further. 

## 3. Experiments and Results

After exploring the methods in the public dataset, we conducted experiments on our own data. We hope that our work can provide a general method to solving pre-impact fall detection, helping researchers to analyze characteristics in other datasets.

### 3.1. Experimental Setup

The data was collected from an inertial sensor (MPU9250) worn on the waist, with y-axis representing the direction of gravity. MPU9250 can detect tri-axial acceleration, tri-axial angular velocity, and three attitude angles with a sampling rate of 50 Hz. Kalman filtering is used to filter the noises in the data. Integrated with a Bluetooth module, the sampled data can be sent to a computer. The data was gathered from nine healthy volunteers (1 female and 8 males, age 26 ± 3 years, height 169.5 ± 9.5 cm, and weight 66.5 ± 16 kg). Each volunteer was required to stage four types of falls (including forward, backward, and two lateral), and four types of activities of daily life (including walking, jogging, standing up, and lying down from a sitting position). For the fall category, volunteers performed simulated fall action from a standing position, and finally impacted onto a 20-cm-thick mattress. For the ADL category, volunteers were instructed to walk and jog on the ground, stand up from a chair, and lie down onto the same mattress from a sitting position. In total, 106 trails of ADLs and 151 trails of falls were collected and labeled manually. 

**Figure 6:**
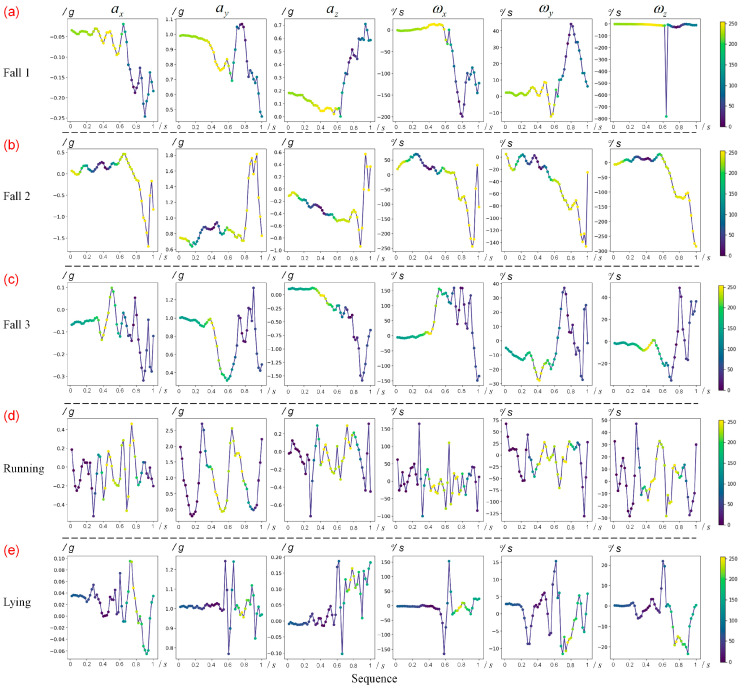
The hot map of five samples of real-world data. The color bar indicates the importance of regions in the sequence for classification. $a_{x}$, $a_{y}$, and $a_{z}$ represent x-axis, y-axis, and z-axis of acceleration, respectively. $\omega_{x}$, $\omega_{y}$, and $\omega_{z}$ represent x-axis, y-axis, and z-axis of angular velocity, respectively. (**a**) One common fall sample. (**b**) One fall sample. Note that the impact phase is also a contributing region of this set. (**c**) One fall sample. Note that the minimum SVM in the early fall phase is less than 0.6 g (g for one gravitational acceleration). (**d**) One running sample. (**e**) One lying sample.

We split the data into train dataset (185 trails) and test dataset (72 trails), and captured the first one-second time window on each activity trail as the input of the CNN. Following the CAM method, we obtained the hot maps of all test samples. Intuitively, based on the contributing region (the highlighting region) on the hot maps, we found that the characteristics of SVM learned by CNN, similar to the results verified on the MobiAct dataset, contributed to identify a pre-impact fall, mostly in the early fall phase. Of course, there are two sets of data showing that the impact phase is also a contributing region, one of which is shown in Figure 6b; while in order to detect the pre-impact fall, the characteristics of the impact phase are not considered here.

### 3.2. Parameter Selection for Pre-Impact Fall Detection

From the principles in Section 2, it can be seen that pre-impact fall detection still has some parameters to decide: feature selection, threshold determination, and the pre-impact detection time window size.

The most important difference between a fall and other activities is the acceleration change in SVM. According to the results of the hot map, the SVM in the early fall phase is generally between 0.6 g and 0.9 g (the few exceptions are shown in Figure 6c), and the SVM on the start frame is generally around 0.95 g (g for one gravitational acceleration). Therefore, we use 0.95 g and 0.6–0.9 g as the thresholds of SVM for the start frame and early fall phase, respectively. Given that the angular velocity is high in some ADLs, such as lying, shown in Figure 6e, the angular velocity is added as another feature, with a threshold of 100°/s.

The aim of the detection time window size was to ensure that the falls are detected during the early fall phase. As shown in hot maps, the number of yellow points is 7–12 in the pre-impact stage, so the slight weightlessness usually lasts 0.14–0.24 s because of the 50 Hz sampling rate. We hoped to pre-detect a fall as soon as possible, leaving a long lead time. While volunteers performed other ADLs, such as walking and running, shown in Figure 6d, the value of SVM also showed a brief decline when volunteers had one foot off the ground, which is similar to the characteristics of fall action. However, in walking and running, the duration of slight weightlessness was relatively short, usually less than 0.1 s. Thus, we chose a pre-impact time window size of 0.4 s. If half of the data in the time window met the thresholds set above, the algorithm detected it as a fall.

### 3.3. Experimental Result

**Figure 7:**
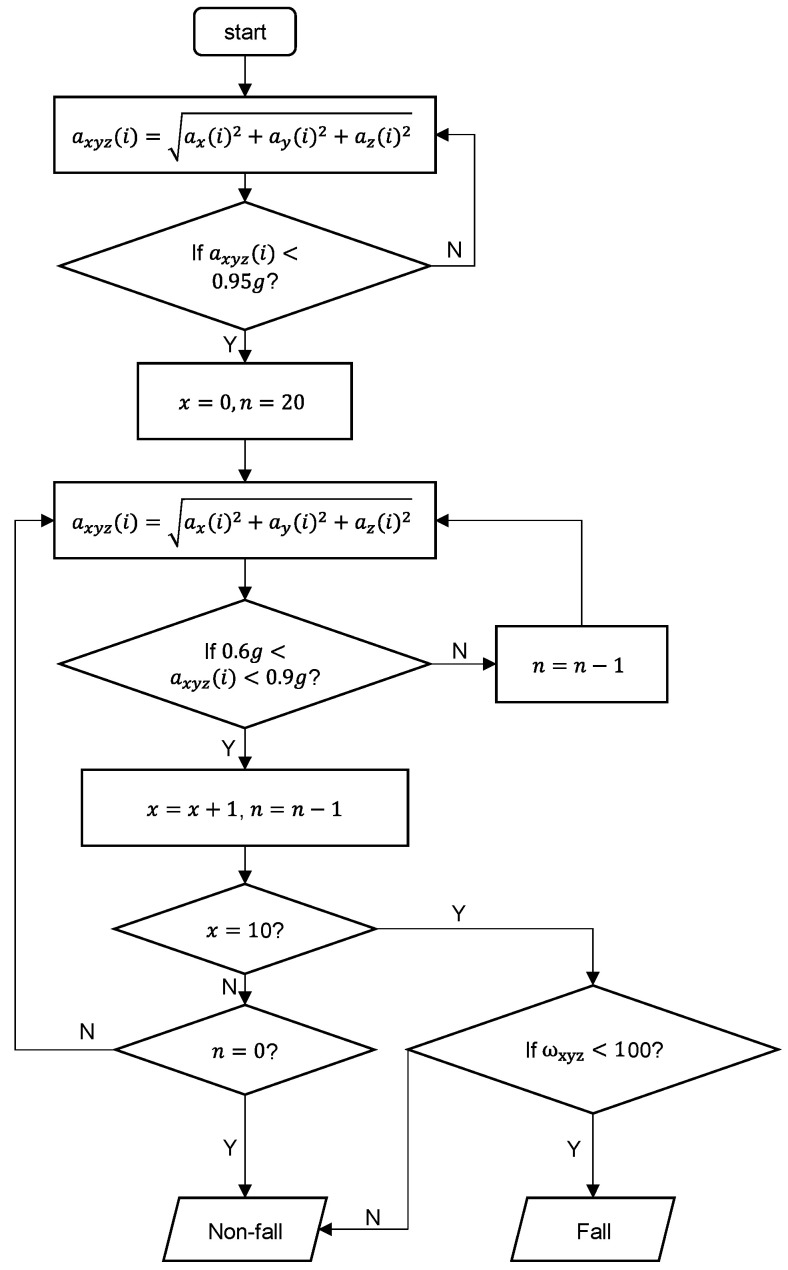
The threshold-based algorithm flow chart.

The threshold-based algorithm flow chart is shown in Figure 7. Sensitivity, specificity, and accuracy were calculated to measure the performance of algorithm. Sensitivity, also called the true positive rate, measures the proportion of falls that are correctly detected as falls. Specificity, also called the true negative rate, measures the proportion of ADLs correctly detected as ADLs. In this study, sensitivity is the extent to which falls are not overlooked, and specificity is the extent to which ADLs are classified as non-falls. Our algorithm correctly identified 142 fall samples and 103 ADL samples, and achieved 95.33%, 94.04%, and 97.17% of accuracy, sensitivity, and specificity, respectively.

### 3.4. Comparison with Other Studies

We compared our threshold-based algorithm with those from some updated studies on pre-impact fall detection. To objectively evaluate the performance, we conducted experiments on the same benchmark. We followed the algorithm flow charts reported in these studies, and tested the methods with our self-collected dataset, including the pre-impact fall data and ADLs data. The performance comparison is shown in Table 1, and our proposed algorithm achieved the highest accuracy.

## 4. Discussion

In the current research of fall detection, some studies used a public dataset for deep learning research to achieve state-of-the-art accuracy. Casilari et al. [18] studied the application of CNNs with multiple public datasets, and the CNN achieved 99.22% accuracy on the SisFall dataset [19]. The CNN-LSTM model reported by Hassan et al. [20] achieved 96.75% accuracy on the MobiAct dataset, which was claimed as a state-of-the-art approach. However, these studies focused on the improvement of accuracy without taking computational cost into account, which made practical application on wearable devices difficult. Moreover, there are few studies on pre-impact fall detection using data from the early fall stage. Casilari et al. [18] performed experiments on the MobiAct dataset, where observation time window of 1s around the peak of acceleration magnitude was used as the input of the CNN. The comparison of performance is shown in Table 2. Our proposed CNN model with the GAP layer not only achieves 95.55% of accuracy during the early fall phase, but also establishes the mapping relationship between feature maps and classes. Thus, we propose our threshold algorithm by analyzing the hot maps of the contribution region in the original data to the classification result. 

While some studies extracted handcrafted features and constructed fixed-threshold algorithms for real-world use, Jung et al. [17] obtained 100% sensitivity, 97.54% specificity, and 98.33% accuracy on their private dataset, and achieved relatively high performance in comparison to some similar studies on the SisFall dataset. However, as shown in Table 1, when evaluating Jung’s algorithm with our experimental data, 87.16% accuracy and 77.36% specificity were achieved, due to the diversity of individuals, behaviors, and environments. The methods reported by Zhao [15] and Ahn [16] also showed a significant decrease in the specificity. The reason is that they used thresholds of one single timestamp, rather than a time window. Jung used acceleration, angular velocity, and vertical angle with thresholds of 0.82 g, 47.3°/s, and 45° as the features to detect a fall. These data characteristics were the features of fall action, but they also existed in other ADLs, such as walking and running, mentioned above. Our threshold algorithm is designed according to the contributing region learned by the CNN. By analyzing the features and characteristics of a time window, our proposed algorithm achieved higher accuracy in comparison with these pre-impact fall detection algorithms.

In some early studies, Noury et al. [21] and Boissy et al. [22] tested fall detection during syncope conditions and near falls, and their methods also achieved a similar performance, reported in Table 1, though their detections were not focusing on the pre-impact phase. In this study, our experimental conditions did not include near falls or syncope fall events for now, and we will improve the experimental conditions to study the tough challenges on specificity in the future work.

## 5. Conclusions

In this study, we propose a pre-impact fall detection approach by applying a machine learning-based method on the data from an IMU-sensor. By integrating the CNN and CAM methods, we obtained hot maps of the fall data, which intuitively demonstrates the contribution of different regions to achieve successful fall detection. After training on the MobiAct dataset, our model could achieve high accuracy. Then, we merged the threshold-based method to detect falls in our real-world data, and the method achieved high-accuracy detection. Since some studies are based on their private datasets, this article aims to provide a general analysis of fall data, rather than comparing accuracy of existing methods. They can also obtain the hot map through the CAM method, and then perform feature extraction and analysis on the data. Although the algorithm proposed in this paper has achieved a high accuracy with our real-world data, there were still a few fall data that do not meet the threshold in the algorithm. In future works, the accuracy will be further improved under more specific feature analysis, and experiments will be conducted on more public datasets.

## Figures and Tables

**Table 1:** Performance comparison with some threshold-based studies.

Method	Zhao et al. [15]	Ahn et al. [16]	Jung et al. [17]	This Study
Accuracy (%)	81.32	83.27	87.16	95.33
Sensitivity (%)	95.36	96.69	94.04	94.04
Specificity (%)	61.32	64.18	77.36	97.17
Feature	Acceleration Angular velocity	Acceleration Angular velocity Vertical angle	Acceleration Angular velocity Vertical angle	Acceleration Angular velocity Time window

**Table 2:** Performance comparison with learning-based studies.

	Hassan et al. [20]	Casilari et al. [18]	This Study
Accuracy (%)	96.75	80.71	95.55
Sensitivity (%)	98.00	49.26	94.52
Specificity (%)	96.00	95.50	96.52
Network model	CNN-LSTM	CNN	CNN
Time window	Full action	1 s	1 s
